# Gastroesophageal reflux disease and its related factors among women of reproductive age: Korea Nurses’ Health Study

**DOI:** 10.1186/s12889-018-6031-3

**Published:** 2018-09-21

**Authors:** Oksoo Kim, Hee Jung Jang, Sue Kim, Hea-Young Lee, Eunyoung Cho, Jung Eun Lee, Heeja Jung, Jiyoung Kim

**Affiliations:** 1 College of Nursing, Ewha Womans University, Seoul, Republic of Korea; 2 Ewha Research Institute of Nursing Science, Seoul, Republic of Korea; 3 Division of Nursing, Hallym University, 1 Hallymdaehak-gil, Chuncheon, 24252 Republic of Korea; 4 Research Institute of Nursing Science, Hallym University, Chuncheon, Republic of Korea; 5 College of Nursing, Yonsei University, Seoul, Republic of Korea; 6 Department of Nursing, Doowon Technical University, Anseong, Republic of Korea; 7 Department of Dermatology, Warren Alpert Medical School, Brown University, Providence, RI USA; 8 Department of Epidemiology, Brown University School of Public Health, Providence, RI USA; 9 Department of Food and Nutrition, College of Human Ecology, Seoul National University, Seoul, Republic of Korea; 10 College of Nursing, Konyang University, Daejeon, Republic of Korea; 11 Department of Nursing, Dong-eui University, Busan, Republic of Korea

**Keywords:** Body mass index, Depression, Gastroesophageal reflux, Nurses, Reproductive age

## Abstract

**Background:**

Recently, the number of patients diagnosed with gastroesophageal reflux disease (GERD) has increased in Korea. Risk factors of GERD include age, sex, medication use, lack of physical exercise, increased psychological stress, low or high body mass index (BMI), unhealthy eating habits, increased alcohol consumption, and cigarette smoking. However, few studies examined the major factors affecting GERD in women of childbearing age. Therefore, this study assessed the risk factors of GERD among 20,613 female nurses of reproductive age using data from the Korea Nurses’ Health Study.

**Methods:**

Participants were recruited from July 2013 to November 2014. They provided their history of GERD 1 year prior to data collection, along with information on their demographic characteristics, health-related behaviors, diet, medical history, and physical and psychological factors. Of the total sample, 1184 individuals with GERD diagnosed in the year prior to the study were identified. Propensity score matching was used for analysis.

**Results:**

Cigarette smoking, increased alcohol consumption, low or high BMI, depression, and increased psychosocial stress were associated with the prevalence of GERD among Korean young women. Multivariate ordinal logistic regression analysis revealed significant positive relationships between GERD and being a former smoker; having a low (< 18.5 kg/m^2^) or high BMI (> 23 kg/m^2^); and having mild, moderate, moderately severe, and severe depression.

**Conclusions:**

Smoking, BMI, and depression were associated with GERD. To reduce this risk among female nurses, intervention strategies are required to help nurses maintain a normal weight and manage their depression.

## Background

Gastroesophageal reflux disease (GERD) occurs when the lower esophageal sphincter relaxes inappropriately, thereby permitting gastric acid to enter the distal esophagus. The most common symptoms associated with GERD are heartburn and acid regurgitation [1]. GERD is a chronic disease that tends to relapse and cause extra-esophageal complications, including aspiration pneumonia, reflux-induced asthma, reflux cough syndrome, and laryngitis [2]. GERD can also lead to esophageal complications such as erosive esophagitis, bleeding and peptic strictures, and chronic GERD increases the risk of Barrett’s esophagus, which can progress to esophageal cancer [2, 3]. Persistent GERD symptoms may also lead to sleep deprivation, lower quality of life, and decreased work productivity [4–6]. In fact, more than 50% of GERD patients experience heartburn when awake [7], and nighttime reflux causes sleep disorders [4]. Therefore, it is important to diagnose and treat GERD before it progresses.

In South Korea, the prevalence of GERD was 7.1% in 2012 among adults aged 20–69 years [8]. Furthermore, the number of patients diagnosed with GERD increased, from 1.99 million in 2008 to 3.36 million in 2012 (an increase of about 69%). Over that same period, the total medical costs related to GERD increased by 50.2% [9]. Women are inordinately represented in this population: female patients with GERD account for as much as 60.3% of adult patients aged less than 65 years [10], and during pregnancy, GERD is reported by 40–85% of women [11]. Women also tend to have a higher frequency of GERD symptoms and lower quality of life when compared to male patients with GERD [1].

Besides sex, the known risk factors of GERD include age, medication use [12–15], lack of physical exercise, increased psychological stress, low or high body mass index (BMI), unhealthy eating habits, increased alcohol consumption, and cigarette smoking [14, 15]. Furthermore, frequent night-shift workers were 1.38 times more likely to develop GERD [16].

Women constitute 97% of the nursing workforce in South Korea, of which the majority are of reproductive age (20–45 years) according to statistical data of license information system in 2016 [17]. Fixed-shift nurses are rare in Korea, with most nurses experiencing rotating shifts on a regular basis, including night shifts. Nurses on rotating shifts with frequent night duties tend to have poor sleep quality, which leads to a lack of physical activity, and higher BMI and poor eating habits [18]. Frequent night work can have a number of other adverse physical and mental health consequences for nurses [19], such as the high prevalence of binge eating found among Korean nurses [20]. Given their night-shift duties, and its association with other health problems, Korean nurses may have a higher risk of developing GERD. However, the prevalence of GERD, and the impact of rotating night-shifts, health behaviors in response to stressful work factors (e.g., irregular diet, smoking, alcohol intake, etc.), BMI, and depressive mood on GERD, have not been fully explored in previous studies.

### Purpose

We investigated GERD-related factors among nurses of reproductive age, including general characteristics, health-related factors, and physical and psychological factors, based on data collected by the Korean Nurses’ Health Study (KNHS). The results of this study can suggest future directions in the promotion of nurses’ health and boost efforts to reduce the prevalence of GERD in this group. We had the following objectives:

1. To identify the prevalence of GERD in Korean nurses of reproductive age.
2. To identify the risk factors (including demographic characteristics, lifestyle behaviors, and psychological factors) associated with GERD in this population.

## Methods

### Study design and sample

The KNHS is the first large-scale time-series cohort study of reproductive-aged women in South Korea. Nurses between 20 and 45 years of age were invited to complete a health survey with protocols and questionnaires quasi-identical to those of the Nurses’ Health Study III in the US. The KNHS was conducted between March 2013 and December 2015 (spanning 3 years) as a web-based questionnaire survey. In the KNHS, each participant was required to complete four time-series online questionnaires (Modules 1–4) consisting of the initial questionnaire and three follow-up questionnaires, delivered in 6-month intervals. The study design and protocol have been described elsewhere [21].

This study was conducted to identify variables influencing GERD among registered nurses. Of the 20,613 nurses aged 20–45 years who participated in the KNHS Module 1 between July 1, 2013 and November 11, 2014, a total of 3151 (15.3%) had been diagnosed with GERD by a physician, of which 1184 (5.7%) were diagnosed within 1 year prior to the survey. To meet the inclusion criteria, the registered nurses had to work in hospitals on a full-time, part-time, or contract basis. The study received institutional review board approval (# 2013-03CON-03-P) from the Korea Centers for Disease Control and Prevention. All nurses signed an electronic written consent before completing the online questionnaire; their anonymity and confidentiality were guaranteed. As the survey was conducted online and respondents were required to answer all questions to be able to continue to the next page, there were no missing data for the respondents included in analysis.

### Measures

The research data were obtained by analyzing the responses collected from participants in the KNHS Module 1. To extract the variables influencing nurses’ GERD, we analyzed the raw data in the factor clusters of demographic characteristics (age, education status, marital status, annual income, and shift work), lifestyle factors (cigarette smoking and alcohol consumption, BMI), and psychological factors (depression and stress). Shift work was included among the demographic variables because it is a characteristic of nurses’ working routine, and thus necessitates adjustment for the participation of women of reproductive age. This is especially important because an association has been found between shift work and GERD [16].

The questionnaire items of the KNHS Module 1, including demographic characteristics, health behavior, illness, medication, family history, pregnancy, mood, employment, occupational exposures, and subjective health perception, were quasi-identical to those of the Nurses’ Health Study III conducted in the US. However, some items were adjusted and supplemented after consultation with an expert panel to enable adaptation to the Korean context. The original version of the questionnaire was translated into Korean and then back-translated into English.

BMI was determined by participants’ reported height in meters and weight in kilograms, and was categorized as low (underweight [< 18.5 kg/m^2^] vs. normal [≥18.5 to < 23 kg/m^2^]) or high (overweight [≥23 to < 25 kg/m^2^] vs. normal) based on previously published criteria [22].

Depression was measured by the Patient Health Questionnaire-9, a self-reported brief depression assessment tool developed to determine depression levels based on the Fourth Edition of the Diagnostic and Statistical Manual of Mental Disorders [23]. It comprises 9 items rated on a 4-point Likert scale: not at all (0), several days (1), more than half the days (2), and nearly every day (3). Total scores range from 0 to 27, and higher scores indicate higher depression levels. A score of zero means no depression, and depression is categorized as minimal (1–4), mild (5–9), moderate (10–14), moderately severe (15–19), and severe depression (20–27). However, for the present analysis, the 0 and 1–4 ranges were grouped together to form a range of 0–4 because a score of zero was not observed in any of the questionnaires analyzed. The Cronbach’s alpha was .90 in our study, which was similar to that observed in the original study (.87) [24].

Stress level was measured using the Perceived Stress Scale-4, which consists of 4 items measured on a 5-point scale (0 = never to 4 = very often) [25]. The total score ranges from 0 to 16, with higher scores indicating higher stress levels. The Cronbach’s alpha in the initial study was .72 and .50 for our study.

### Data analysis

The data were analyzed using SPSS Statistics 22.0. The demographic variables, including shift work, were matched to ensure no differences in frequency between participants diagnosed with GERD within 1 year prior to the survey (*n* = 1184) and non-GERD participants. Differences between the GERD and non-GERD groups regarding demographic characteristics, lifestyle factors, and psychological factors, were analyzed through the chi-square test and *t*-test. To reduce bias from distorted samples between GERD and non-GERD groups [26], the 1:1 propensity score matching (PSM) method was employed, after controlling for demographic variables (including shift work). This was followed by multivariate ordinal logistic regression analysis to examine the factors influencing GERD.

To identify the factors associated with GERD, demographic characteristics, lifestyle factors, and psychological factors were used as independent variables in the following three models. Analysis of odds ratios was done to find the factors influencing reflux esophagitis.

- Model 1: demographic characteristics (age, educational status, marital status, annual income, and shift work).
- Model 2: demographic characteristic (age, educational status, marital status, annual income, and shift work) plus lifestyle factors (cigarette smoking, alcohol consumption, and BMI).
- Model 3: demographic characteristic (age, educational status, marital status, annual income, and shift work), plus lifestyle factors (cigarette smoking, alcohol consumption, and BMI), plus psychological factors (depression and stress).

## Results

### Sample characteristics

The demographic and shiftwork characteristics of the sample after PSM are shown in Table 1. Roughly 43% of the respondents were in their 30s, while 36% were in their 20s. More than half (62%) were non-married and 69% worked rotating schedules that included night shifts.

**Table 1:** Demographic characteristics of GERD and non-GERD groups after propensity score matching n(%)

Variables	GERD (*n* = 1184)	Non-GERD (*n* = 1184)	Total (*N* = 2368)	*t*/χ^2^	*p*-value
Age (years)					
≤ 29	433 (36.6)	426 (36.0)	859 (36.3)	.11	.949
30–39	505 (42.6)	512 (43.2)	1017 (42.9)		
≥ 40	246 (20.8)	246 (20.8)	492 (20.8)		
Educational status					
3-year college	533 (45.0)	506 (42.7)	1039 (43.9)	1.28	.528
4-year college	538 (45.5)	558 (47.1)	1096 (46.3)		
Master’s degree or higher	113 (9.5)	120 (10.2)	233 (9.8)		
Marital status					
Single	732 (61.8)	728 (61.5)	1460 (61.7)	.03	.986
Married	443 (37.4)	447 (37.7)	890 (37.5)		
Divorced or widowed	9 (.8)	9 (.8)	18 (.8)		
Annual income (USD)					
≤ 20,000	35 (3.0)	24 (2.0)	59 (2.5)	3.29	.511
20,000–29,999	445 (37.6)	440 (37.2)	885 (37.3)		
30,000–39,999	439 (37.1)	436 (36.8)	875 (37.0)		
40,000–49,999	158 (13.3)	178 (15.0)	336 (14.2)		
≥ 50,000	107 (9.0)	106 (9.0)	213 (9.0)		
Work schedule					
Non-rotating (no night shift)	368 (31.1)	350 (29.6)	718 (30.3)	.65	.421
Rotating with night shift	816 (68.9)	834 (70.4)	1650 (69.7)		

Note: *GERD* gastroesophageal reflux disease

The variables that exhibited significant differences according to GERD status were cigarette smoking, BMI, and depression (Table 2). A statistically significant difference was also shown in stress levels between the GERD (M = 6.65, SD = 2.35) and non-GERD groups (M = 6.35, SD = 2.40; *p* = .002).

**Table 2:** Distribution of risk factors among GERD and non-GERD groups after matching n (%)

Variables	GERD (*n* = 1184)	Non-GERD (*n* = 1184)	Total (*N* = 2368)	*t*/χ^2^	*p*-value
Cigarette smoking					
Non-smoker	1128 (95.2)	1157 (97.7)	2285 (96.5)	10.56	.005
Former smoker	40 (3.4)	20 (1.7)	60 (2.5)		
Current smoker	16 (1.4)	7 (0.6)	23 (1.0)		
Alcohol consumption					
Non-drinker	112 (9.5)	126 (10.6)	238 (10.1)	1.72	.633
Less than 1/month	598 (50.5)	608 (51.4)	1206 (50.9)		
2–4/month	339 (28.6)	316 (26.7)	655 (27.7)		
More than 2/week	135 (11.4)	134 (11.3)	269 (11.3)		
BMI					
Normal	731 (61.7)	810 (68.4)	1541 (65.1)	11.69	.003
Underweight	218 (18.4)	176 (14.9)	394 (16.6)		
Overweight	235 (19.9)	198 (16.7)	433 (18.3)		
Depression					
Minimal	340 (28.8)	440 (37.2)	780 (32.9)	30.12	<.001
Mild	448 (37.8)	453 (38.3)	901 (38.1)		
Moderate	224 (18.9)	177 (14.9)	401 (16.9)		
Moderately severe	121 (10.2)	80 (6.8)	201 (8.5)		
Severe	51 (4.3)	34 (2.8)	85 (3.6)		

Note: *BMI* body mass index

### Factors associated with GERD

Results of the odds ratio analyses are presented in Table 3 for the three models compared, which found that model 3 was most significant and the odds of GERD were higher among former smokers than among non-smokers. Furthermore, these odds among underweight and overweight participants were higher than were those among participants with a normal BMI. Finally, the odds of GERD among participants with mild, moderate, moderately severe, and severe depression were higher than were those among participants with minimal depression.

**Table 3:** Odds ratio and 95% confidence interval for GERD in the multivariate ordinal logistic regression (*N* = 2368)

Variables	Model 1	Model 2	Model 3
	OR (95%CI)	OR (95%CI)	OR (95%CI)
Age (years)			
29≥	Ref	Ref	Ref
30–39	1.02(0.83–1.25)	1.02(0.83–1.26)	1.05(0.85–1.30)
40≤	1.04(0.76–1.42)	1.06(0.77–1.45)	1.17(0.85–1.61)
Educational status			
3-year college	Ref	Ref	Ref
4-year college	0.92(0.77–1.09)	0.93(0.77–1.11)	.93(0.78–1.12)
Master’s degree or higher	0.88(0.63–1.23)	0.90(0.65–1.26)	.91(0.65–1.27)
Marital status			
Single	Ref	Ref	Ref
Married	0.99(0.80–1.22)	0.98(0.79–1.22)	1.04(0.84–1.30)
Divorced or widowed	0.96(0.37–2.50)	1.03(0.39–2.69)	1.05(0.40–2.76)
Annual income (USD)			
20,000≥	Ref	Ref	Ref
20,000–29,999	0.69(0.41–1.19)	0.70(0.41–1.21)	0.76(0.44–1.31)
30,000–39,999	0.70(0.41–1.21)	0.71(0.41–1.22)	0.76(0.44–1.32)
40,000–49,999	0.63(0.35–1.12)	0.63(0.35–1.12)	0.68(0.38–1.23)
50,000≤	0.71(0.38–1.32)	0.72(0.38–1.34)	0.79(0.42–1.48)
Shift work			
No	Ref	Ref	Ref
Yes	0.92(0.76–1.11)	0.92(0.76–1.11)	0.87(0.72–1.06)
Cigarette smoking			
Non-smoker		Ref	Ref
Former smoker		1.93^*^(1.12–3.35)	1.89^*^ (1.08–3.28)
Current smoker		2.31 (0.94–5.66)	2.01(0.82–4.98)
Alcohol consumption			
Non-drinker		Ref	Ref
Below 1/month		1.11(0.84–1.48)	1.11(0.84–1.48)
2–4/month		1.23(0.91–1.66)	1.25(0.92–1.69)
Over 2/week		1.11(0.78–1.58)	1.11(0.78–1.58)
BMI			
Normal		Ref	Ref
Underweight		1.38^**^(1.10–1.73)	1.38^**^(1.10–1.74)
Overweight		1.31^*^(1.06–1.64)	1.32^*^(1.06–1.64)
Depression			
Minimal			Ref
Mild			1.33^**^(1.08–1.63)
Moderate			1.67^**^(1.28–2.17)
Moderately severe			1.97^**^(1.39–2.80)
Severe			1.97^**^(1.19–3.26)
Stress			1.01(0.97–1.05)
Nagelkerke R^2^	0.003	0.016	0.033
χ^2^/df	1.597/8	10.229/8	6.802/8

Note: *OR* odds ratio, *CI* confidence interval, *BMI* body mass index, ^*^p < .05; ^**^p < .01

Model 1: demographic characteristics (age, sex, educational status, marital status, annual income, shift work); Model 2: demographic characteristics and lifestyle factors (cigarette smoking, alcohol consumption, and BMI); Model 3: demographics, lifestyle factors, and psychological factors (depression, stress)

## Discussion

In this study, the estimated prevalence of GERD diagnosed within 1 year prior to the survey among nurses aged 24–45 years was 5.7%. This is slightly higher than the reported rate of 4.8% among Korean women in 2007 [27], In the general Korean population aged 20–69 years, the prevalence of GERD was reported as 7.1% in 2012, although it did not differentiate rates by gender [8]. Our finding is also similar to the rate (5.2–8.5%) across East Asia in general over the past decade (2005–2015), as found in a systematic review [28].

The factors found to be independently associated with GERD were cigarette smoking history, being underweight or overweight, and having depression. Interestingly, for smoking history, we found a statistically significant association for former smoking, but no association for current smoking or never smoking. These results might be partly due to the small number of current smokers (1.4%) in our study sample. Several epidemiologic studies have similarly reported that cigarette smoking (i.e., at least one pack per day or habitual smoking) is a risk factor for GERD in East Asian populations [12–15, 28]. Furthermore, this study found that former smokers (*n* = 40, 3.4%) had a significantly higher risk of GERD compared to those who never smoked (*n* = 1128, 95.2%). While former smoking in women should be an important consideration, future studies need to further explore the role of current smoking in GERD among reproductive women.

The results concerning alcohol drinking in our study conflict with those of previous studies. In our study, roughly half of respondents reported drinking less than once per month, and alcohol consumption was not associated with GERD prevalence. In contrast, previous studies identified that a drinking frequency of at least once a week within the past year was a risk factor for GERD [13, 14]. This should be clarified in further prospective studies.

Obesity has previously been found to be a strong risk factor for GERD [29]; however, limited evidence exists for Asian populations, possibly because of the relatively smaller variance in BMI among members of the Asian population compared to among Western populations. The distribution of BMI categories in our sample of Korean nurses was as follows: normal (61.7%), overweight (19.8%), and underweight (18.4%). We found that both underweight and overweight were associated with higher odds of GERD, with underweight having a more substantial influence than overweight. Previous studies also identified BMI as an important factor for GERD prevalence among Japanese participants. However, because these studies targeted both sexes, the differences in prevalence were not as high as those in our study, given that women have a lower BMI increase rate than men [30]. The prevalence of underweight was generally high in our sample, possibly because it included only female nurses, People may become underweight after developing GERD because it limits food consumption or cause loss of appetite. In contrast, the observed association with overweight might be due to weight gain: even moderate weight gain among persons of normal weight can cause or exacerbate reflux symptoms [31]. Unfortunately, the cross-sectional nature of our study prevents us from clarifying these potential causal associations.

We found that the odds of GERD increased with increasing levels of self-reported depression. Past studies have indicated that GERD causes depression [32] and that depression is associated with an increased risk of GERD [33]. One Chinese study found that patients with GERD often exhibit depression, and that those with severe depression tend to require intensive GERD treatment [34]. These findings are consistent with those of our study. Again, although our study could not infer a causal relationship because of its cross-sectional nature, our findings suggest a potential link between depression and GERD risk among Korean women.

Our study has several strengths. First, the sample was large (*N* = 20,613), which allowed us to identify more than 1100 nurses who had been diagnosed with GERD within the year prior to the study. Second, registered nurses tend to report medication use and history of disease with higher accuracy than do the general population, which may support higher accuracy of our data.

As for study limitations, we failed to control for all relevant variables, such as the type and quantity of intake of food (e.g., coffee, instant foods), and did not have access to participants’ medical records. These failures are a result of the limitations of collecting data through national surveys. Also, a self-reported questionnaire was used to obtain disease diagnosis.

Based on these limitations, we recommend that the following be considered in future research:

- The results of blood tests and endoscopies are encouraged to improve the accuracy of disease diagnosis in GERD studies.
- Continuous follow-up of the interplay among lifestyle factors, depressive mood, and GERD changes until and beyond middle age would be beneficial, as GERD risk has been reported to be associated with middle-age in Koreans [35].

## Conclusions

Our investigation of factors associated with GERD prevalence among nurses of reproductive age revealed that a history of past smoking, being underweight and overweight, and depression status are risk factors for GERD. These findings highlight that, to lower GERD prevalence among women of reproductive age, it is necessary to develop measures to monitor former smokers, and to assist overweight and underweight women in achieving and maintaining a normal weight. Furthermore, the identified factors should be considered during the assessment of GERD symptoms.

The findings also have implications for maternal health. Around 5.7% of women of reproductive age reported being diagnosed with GERD in the past year, and their symptoms are likely to worsen during pregnancy. Accordingly, more comprehensive studies on the health of working young women and proactive management of GERD before pregnancy are needed. Accessible programs within the workplace that can aid weight management and alleviate depressive mood are needed, as are further prospective studies on not only nurses, but also a more diverse population of young working women.

## Abbreviations

BMI: Body mass index
GERD: Gastroesophageal reflux disease
KNHS: Korea Nurses’ Health Study

## References

[1] Lee SW, Lien HC, Lee TY, Yang SS, Yeh HJ, Chang CS (2014). Heartburn and regurgitation have different impacts on life quality of patients with gastroesophageal reflux disease. World J Gastroenterol.

[2] Mikami DJ, Murayama KM (2015). Physiology and pathogenesis of gastroesophageal reflux disease. Surg Clin North Am.

[3] Parasa S, Sharma P (2013). Complications of gastro-oesophageal reflux disease. Best Pract Res Clin Gastroenterol.

[4] Fass R (2010). Effect of gastroesophageal reflux disease on sleep. J Gastroenterol Hepatol.

[5] Suzuki H, Matsuzaki J, Masaoka T, Inadomi JM (2014). Greater loss of productivity among Japanese workers with gastro-esophageal reflux disease (GERD) symptoms that persist vs resolve on medical therapy. Neurogastroenterol Motil.

[6] Tack J, Becher A, Mulligan C, Johnson DA (2012). Systematic review: the burden of disruptive gastro-oesophageal reflux disease on health-related quality of life. Aliment Pharmacol Ther.

[7] Jha LK, Maradey-Romero C, Gadam R, Hershcovici T, Fass OZ, Quan SF (2015). The effect of antireflux treatment on the frequency of awakenings from sleep in patients with gastroesophageal reflux disease. Neurogastroenterol Motil.

[8] Min BH, Huh KC, Jung HK, Yoon YH, Choi KD, Song KH (2014). Functional dyspepsia study Group of Korean Society of Neurogastroenterology and motility. Prevalence of uninvestigated dyspepsia and gastroesophageal reflux disease in Korea: a population-based study using the Rome III criteria. Dig Dis Sci.

[9] Ministry of Health and Welfare. Health insurance review & assessment service. GERD medical personnel and expenses. 2013. http://www.mohw.go.kr/front_new/al/sal0301vw.jsp?PAR_MENU_ID=04&MENU_ID=0403&page=137&CONT_SEQ=284315&SEARCHKEY=DEPT_NM. Accessed 28 Sept 2017.

[10] Lee S-W, Chang C-M, Chang C-S, Kao A-W, Chou M-C (2011). Comparison of presentation and impact on quality of life of gastroesophageal reflux disease between young and old adults in a Chinese population. World J Gastroenterol.

[11] Ali RA, Egan LJ (2007). Gastroesophageal reflux disease in pregnancy. Best Pract Res Clin Gastroenterol.

[12] Fujiwara Y, Kubo M, Kohata Y, Machida H, Okazaki H, Yamagami H (2011). Cigarette smoking and its association with overlapping gastroesophageal reflux disease, functional dyspepsia, or irritable bowel syndrome. Intern Med.

[13] Lee SJ, Jung MK, Kim SK, Jang BI, Lee SH, Kim KO (2011). Clinical characteristics of gastroesophageal reflux disease with esophageal injury in Korean: focusing on risk factors. Korean J Gastroenterol.

[14] Matsuki N, Fujita T, Watanabe N, Sugahara A, Watanabe A, Ishida T (2013). Lifestyle factors associated with gastroesophageal reflux disease in the Japanese population. J Gastroenterol.

[15] Yamamichi N, Mochizuki S, Asada-Hirayama I, Matsuda RM, Shimamoto T, Konno-Shimizu M (2012). Lifestyle factors affecting gastroesophageal reflux disease symptoms: a cross-sectional study of healthy 19864 adults using FSSG scores. BMC Med.

[16] Li YM, Du J, Zhang H, Yu CH (2008). Epidemiological investigation in outpatients with symptomatic gastroesophageal reflux from the Department of Medicine in Zhejiang Province, East China. J Gastroenterol Hepatol.

[17] Ministry of Health and Welfare. Health and Welfare Statistical Yearbook. Sejong; 2017.

[18] Son YJ, Park YR (2011). Relationships between sleep quality, fatigue and depression on health promoting behavior by shift-work patterns in university hospital nurses. J Korean Biol Nurs Sci.

[19] Kim HK, Lee TY, Kim KH (2010). The effects of health promotion behavior of shifting nurses’ on the health conditions. J Korea Acad-Industr Coop Soc.

[20] Kim O, Kim MS, Kim J, Lee JE, Jung H (2018). Binge eating disorder and depressive symptoms among females of child-bearing age: the Korea Nurses’ health study. BMC Psychiatry.

[21] Kim OS, Ahn YJ, Lee HY, Jang HJ, Kim S, Lee JE (2017). The Korea Nurse’s health study: a prospective cohort study. J Women’s Health (Larchmt).

[22] Kim MK, Lee WY, Kang JH, Kang J-H, Kim BT, Kim SM (2014). 2014 clinical practice guidelines for overweight and obesity in Korea. Endocrinol Metab.

[23] American Psychiatric Association (2000). Diagnostic and statistical manual of mental disorders, 4th ed., text rev.

[24] Arroll B, Goodyear-Smith F, Crengle S, Gunn J, Kerse N, Fishman T (2010). Validation of PHQ-2 and PHQ-9 to screen for major depression in the primary care population. Ann Fam Med.

[25] Cohen S, Kamarck T, Mermelstein R (1983). A global measure of perceived stress. J Health Soc Behav.

[26] Kim OS, Kim MS, Lee JE, Jung H (2016). Night-eating syndrome and the severity of self-reported depressive symptoms from the Korea Nurses’ health study: analysis of propensity score matching and ordinal regression. Public Health.

[27] Cho SH, Kim CW (2007). The relationship between obesity and reflux esophagitis in health check-up subjects. J Obes Metab Syndr.

[28] Jung HK (2011). Epidemiology of gastroesophageal reflux disease in Asia: a systematic review. J Neurogastroenterol Motil.

[29] El-Serag HB, Graham DY, Satia JA, Rabeneck L (2005). Obesity is an independent risk factor for GERD symptoms and erosive esophagitis. Am J Gastroenterol.

[30] Adachi K, Mishiro T, Tanaka S, Hanada K, Kinoshita Y (2015). Gender differences in the time-course changes of reflux esophagitis in Japanese patients. Intern Med.

[31] Jacobson BC, Somers SC, Fuchs CS, Kelly CP, Camargo CA (2006). Body-mass index and symptoms of gastroesophageal reflux in women. N Engl J Med.

[32] You ZH, Perng CL, Hu LY, Lu T, Chen PM, Yang AC (2015). Risk of psychiatric disorders following gastroesophageal reflux disease: a nationwide population-based cohort study. Eur J Intern Med.

[33] Yang XJ, Jiang HM, Hou XH, Song J (2015). Anxiety and depression in patients with gastroesophageal reflux disease and their effect on quality of life. World J Gastroenterol.

[34] Wong WM, Lai KC, Lam KF, Hui WM, Hu WH, Lam CL (2003). Prevalence, clinical spectrum and health care utilization of gastro-oesophageal reflux disease in a Chinese population: a population-based study. Aliment Pharmacol Ther.

[35] Kim KM, Cho YK, Bae SJ, Kim DS, Shim KN, Kim JH (2012). Prevalence of gastroesophageal reflux disease in Korea and associated health-care utilization: a national population-based study. J Gastroenterol Hepatol.

